# Inferring Evolutionary Timescale of Omsk Hemorrhagic Fever Virus

**DOI:** 10.3390/v15071576

**Published:** 2023-07-19

**Authors:** Artem N. Bondaryuk, Olga I. Belykh, Evgeny I. Andaev, Yurij S. Bukin

**Affiliations:** 1 Laboratory of Natural Focal Viral Infections, Irkutsk Antiplague Research Institute of Siberia and the Far East, Irkutsk 664047, Russia; ui.artem.ui@gmail.com (A.N.B.); e.andaev@gmail.com (E.I.A.); 2 Limnological Institute, Siberian Branch of the Russian Academy of Sciences, Irkutsk 664033, Russia; bukinyura@mail.ru

**Keywords:** substitution rate, evolutionary rate, population dynamics, phylogenetics, temporal signal

## Abstract

Until 2020, there were only three original complete genome (CG) nucleotide sequences of Omsk hemorrhagic fever virus (OHFV) in GenBank. For this reason, the evolutionary rate and divergence time assessments reported in the literature were based on the E gene sequences, but notably without temporal signal evaluation, such that their reliability is unclear. As of July 2022, 47 OHFV CG sequences have been published, which enables testing of temporal signal in the data and inferring unbiased and reliable substitution rate and divergence time values. Regression analysis in the TempEst software demonstrated a stronger clocklike behavior in OHFV samples for the complete open reading frame (ORF) data set (R^2^ = 0.42) than for the E gene data set (R^2^ = 0.11). Bayesian evaluation of temporal signal indicated very strong evidence, with a log Bayes factor of more than 5, in favor of temporal signal in all data sets. Our results based on the complete ORF sequences showed a more precise OHFV substitution rate (95% highest posterior density (HPD) interval, 9.1 × 10^−5^–1.8 × 10^−4^ substitutions per site per year) and tree root height (416–896 years ago) compared with previous assessments. The rate obtained is significantly higher than tick-borne encephalitis virus by at least 3.8-fold. The phylogenetic analysis and past population dynamics reconstruction revealed the declining trend of OHFV genetic diversity, but there was phylogenomic evidence that implicit virus subpopulations evolved locally and underwent an exponential growth phase.

## 1. Introduction

Omsk hemorrhagic fever virus (OHFV) is a human-pathogenic tick-borne flavivirus belonging to the family *Flaviviridae*, genus *Flavivirus*. OHFV is transmitted mainly by *Dermacentor* sp., but despite this, humans are mostly infected by contact with muskrats (*Ondatra zibethicus*), which induces severe hemorrhagic fever [1,2]. In the USSR, more than 1000 cases of Omsk hemorrhagic fever were registered between 1946 and 1958. In 1988, OHFV foci reactivated and the disease reemerged [3]. Since 2022, no cases of Omsk hemorrhagic fever were observed [1]. For a long time, OHFV was detected in the relatively narrow area of Western Siberia (Russia) [3]. But recently, OHFV has been identified outside the Russian Federation in the Republic of Kazakhstan [4].

For more than 17 years, from 2003 to 2020, only five OHFV complete genomes (CG) have been available in GenBank, while three of them are sequences of the prototype strain Bogolubovska. For this reason, the evolutionary rate and divergence time of OHFV reported in the literature were based on partial or complete E gene sequences [3]. The temporal structure or temporal signal of E gene data sets were not evaluated, which calls into question the reliability of OHFV rates and dates reported. From 2020 to 2021, 17 heterochronous (i.e., collected at different time points) OHFV CG sequences were submitted [2]. Kovalev et al. (2021) [2] first provided intriguing insights into the OHFV genetic diversity (in particular, CG sequences of three OHFV subtypes were reported for the first time) and molecular variability using the complete genome data; however, molecular clock analysis that inferred the evolutionary timescale of OHFV was not performed. In March 2022, 25 OHFV CG sequences were deposited, but they all were collected in December 2007 (isochronous samples).

The aim of this study is the evaluation of temporal signal in CG and E gene data available in GenBank, followed by the estimation of OHFV’s evolutionary rate and divergence times. In addition, we reconstructed past population dynamics of OHFV and compared the results with epidemiological data on epizooty in muskrats in Western Siberia since 1945.

## 2. Materials and Methods

### 2.1. Searching Nucleotide Data and Assessing Phylo-Temporal and Population Structures

Nucleotide sequences of OHFV ORF and E genes with known collection dates were searched in the NCBI database using a rentrez package [5] implemented in R, with duplicates being deleted. Totally, 81 E and 43 ORF gene sequences were found. All samples were collected from four adjacent regions of Western Siberian of Russia—the Omsk, Novosibirsk, Kurgan, and Tyumen regions. A total of 25 of 43 ORF sequences were isolated in December 2007 at Tenis Lake (Omsk Region), resulting in both population and phylo-temporal structures; therefore, each data set was subdivided into subsets with (ORF_het+iso_ and E_het+iso_) and without (ORF_het_ and E_het_) the isochronous samples from Tenis Lake to analyze them separately.

### 2.2. Temporal Signal Evaluation and Molecular Clock Model Selection

To examine temporal signal or temporal structure in each nucleotide subset, we used TempEst v1.5.3 [6]. Potentially problematic outliers (i.e., samples with sequencing errors or long passage history) were removed (Figure S1). The total number of samples in each subset after the filtration can be seen in Table 1.

An informal root-to-tips regression analysis in TempEst tends to give false negative results [7] in the case of an ancient root relative to a narrow sampling window. As a formal statistical test of temporal structure, we employed a Bayesian evaluation of temporal signal (BETS) [7] where log marginal likelihoods were estimated with a path-sampling (PS) approach implemented in BEAST v2.6.7 [8] (Table 2). This analysis involves comparing models with and without collection dates associated with the genomes (a test of ultrametricity of the phylogenetic tree). For PS analysis, the pre-burning MCMC length was chosen as 1 × 10^7^, and the number of path steps was 200, with an MCMC length of 5 × 10^5^. A log Bayes factor (difference between log marginal likelihoods of two models) of at least 5 indicates very strong support for one model over another one, a value of 3 indicates strong support, and a value of 1 is considered to be positive evidence.

Selection between a strict clock (SC) and an uncorrelated relaxed clock with an underlying lognormal prior distribution (UCLD) of substitution rates was conducted according to log marginal likelihoods estimated. As a population model, a parametric constant size model with proper priors integrating to 1 was used.

### 2.3. Phylogenetic Analysis

For the visual assessment of phylo-temporal and population structures, a maximum likelihood tree was reconstructed with IQTREE v.1.6.12 [9]. A best-fit substitution model was chosen based on the Bayesian information criterion (BIC) calculated in IQTREE with ModelFinder v2.0 [10].

Bayesian phylogenetic analysis was conducted in BEAST v2.6.7. Substitution models were chosen based on BIC. As a population model, we used a flexible non-parametric Bayesian skyline model [11].

In the main BEAST analyses, the MCMC length varied between 5 × 10^7^ and 1 × 10^8^ iterations, with a sampling frequency at which the total number of trees was at least 40,000. Effective sample sizes (ESS) for all parameters were more than 200 independent samples. The BEAST xml files and logs can be found in the Supplementary Materials.

### 2.4. Isochronous Clade Analysis

The 25 isochronous OHFV samples from Tenis Lake were analyzed in BEAST separately from the other subsets due to their strong population and phylo-temporal structures. Model selection was conducted the same way as it was described above, except for the use of a molecular clock, for which we chose SC as a priori. Based on the analysis of the ORF_het_ data set, the substitution rate was fixed as 1.3 × 10^−4^ substitutions per site per year (s/s/y).

## 3. Results

### 3.1. Structure of Genomic Data Sets

In total, 43 CG and 75 E gene unique nucleotide sequences of OHFV with known collection dates were found in GenBank. The sampling window for both data sets was 60.9 years (1947–2007). For the subsequent phylogenetic analysis, we extracted an open reading frame (ORF) region from the CG data set.

**Figure 1:**
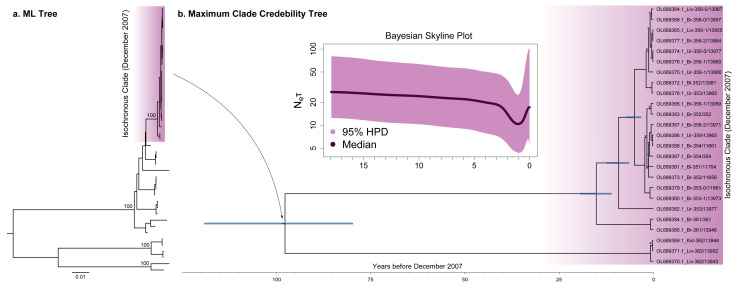
(**a**) Maximum likelihood (ML) tree reconstructed in IQTREE using 43 open reading frame (ORF) sequences (10.245 nt) of OHFV; bootstrap support values of the main nodes based on the 1000 replications are displayed to the left of the nodes. A total of 25 sequences isolated in December 2007 were marked as purple gradient. (**b**) Maximum clade credibility (MCC) tree of the isochronous clade inferred using the fixed substitution rate (1.3 × 10^−4^ substitutions per site per year, s/s/y) from the molecular clock analysis of the ORF_het_ data set. The blue horizontal bars are the 95% highest posterior intervals (HPD) of main node heights. The nodes marked with asterisks have a posterior probability (pp) lower than 0.95. The inner box depicts the OHFV population dynamics for the isochronous clade revealed by a Bayesian skyline plot model with the *Y*-axis in log scale. The *Y*-axis is relative genetic diversity—N_e_ × τ—where N_e_ is an effective population size or, in the context of infectious diseases, it is the effective number of infections [14], and τ is the generation time or time between successive infections in transmission chains; the *X*-axis is time before December 2007.

Preliminary phylogenetic analysis of the ORF data set in IQTREE showed that 25 sequences isolated in December 2007 from a relatively narrow location (Tenis Lake, Omsk Region, Russia) form a monophyletic clade (Figure 1a), which produces phylo-temporal [12] and population structures [13]. Therefore, we decided to subdivide ORF and E gene heterochronous data sets into subsets. In one case, they included the isochronous samples, (ORF_het+iso_ and E_het+iso_), where subscript ‘het’ stands for heterochronous and ‘iso’ for isochronous. The second strategy consisted of excluding isochronous samples, ORF_het_ and E_het_. We analyzed each subset separately. To maximize the level of temporal signal, one random sequence from the isochronous clade was included in the ORF_het_ and E_het_ data sets.

### 3.2. Level of Temporal Signal in the ORF and E Gene Data Sets

All four data sets were tested in TempEst, with the samples furthest from a regression line (outliers) excluded. Results are presented in Table 1.

Despite the larger number of sequences, the E_het_ and E_het+iso_ data sets contained weaker temporal signal (R^2^ is 0.11 and 0.21, respectively) and about six-times fewer parsimony-informative sites. In both ORF and E gene data sets, the isochronous component increased R^2^ values. The higher R^2^ was observed for ORF data sets (0.42 and 0.62 for ORF_het_ and ORF_het+iso_, respectively), which indicated a stronger temporal signal.

Since TempEst implies informal regression assessment, we employed formal BETS analysis. In all data sets, a model with an UCLD clock in which the data are accompanied by the actual collection dates (UCLD_dates_) yielded the highest log marginal likelihood values with very strong support (log Bayes factor of more than 5) (Table 2).

According to the results, we employed molecular clock analysis in BEAST with all four data sets.

### 3.3. Substitution Rate and Tree Root Height with Confidence under Relaxed Clocks

For each subset, the best molecular clock model (SC/UCLD) was chosen according to the highest log marginal likelihood estimated with a path-sampling method (Table 2).

For all data sets, the log Bayes factors of more than 3 (ORF_het_, ORF_het+iso_, E_het_) and 5 (E_het+iso_) indicated strong and very strong support for a UCLD model, respectively, despite the fact that the 95% HPD intervals in all cases included 0.

**Figure 2:**
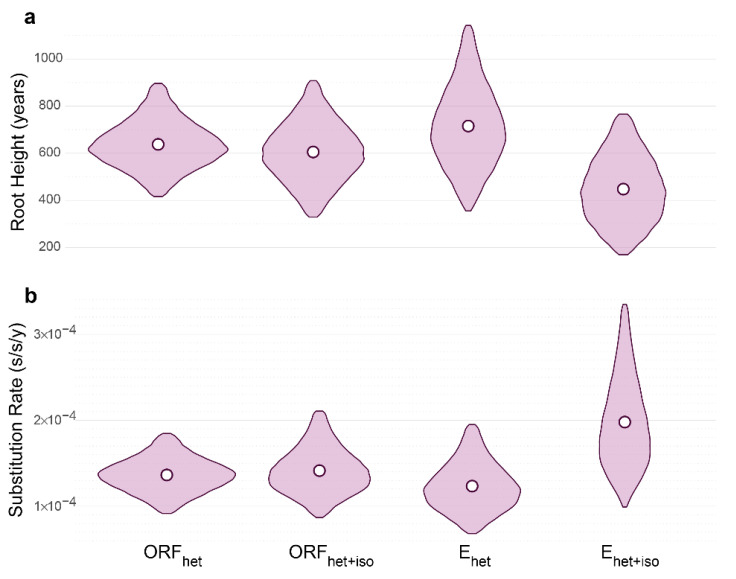
(**a**,**b**) Violin plots of substitution rates and root heights of four Omsk hemorrhagic fever virus (OHFV) data sets. Rates are measured as substitutions per site per year (s/s/y), root ages as years before the most recent sample collected in December 2007. Violin plots represent 95% highest posterior density (HPD), white circles—median values.

The analysis of substitution rates in BEAST demonstrated that in the case of the ORF_het_ (9.1 × 10^−5^–1.8 × 10^−4^ substitutions per site per year, s/s/y), ORF_het+iso_ (8.6 × 10^−5^–2.1 × 10^−4^ s/s/y), and E_het+iso_ (9.9 × 10^−5^–3.3 × 10^−4^ s/s/y) data sets, most of the posterior mass was higher than 1.0 × 10^−4^ s/s/y (Figure 2).

The median values of the ORF_het_ (1.3 × 10^−4^ s/s/y), ORF_het+iso_ (1.3 × 10^−4^ s/s/y), and E_het_ (1.2 × 10^−4^ s/s/y) data sets were similar and lower than E_het+iso_ (1.9 × 10^−4^ s/s/y).

Interestingly, both E gene data sets had the shift of 95% HPD interval but in an opposite direction. So, the lower boundary of E_het_ was 6.8 × 10^−5^ s/s/y, which led to more ancient root height values (more than 1000 years). Conversely, the upper boundary of the E_het+iso_ data set was more than 3.0 × 10^−4^ s/s/y, followed by the youngest OHFV root age with values lower than 200 years. Thus, the E_het+iso_ data set was more affected by the isochronous compound within it. Despite low R^2^ (0.11), E_het_, in turn, showed similar median values, but 95% HPD intervals for roots and rates were wider (Table 1), which indicated less precise estimates.

### 3.4. OHFV Dated Phylogenies and Population Dynamics

To infer the maximum clade credibility (MCC) tree and past population dynamics of OHFV, we analyzed the ORF_het_ data set, as it demonstrated the narrowest 95% HPD intervals for root height and substitution rate values and did not contain a phylo-temporal and explicit population structure. Besides, we also analyzed the isochronous clade separately using the substitution rate deduced from the ORF_het_ analysis.

#### 3.4.1. ORF Data Set without the Isochronous Clade

**Figure 3:**
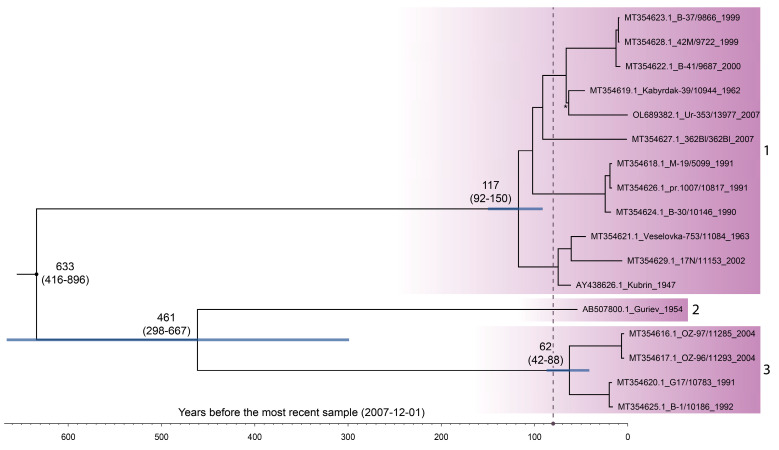
Maximum clade credibility tree of Omsk hemorrhagic fever virus (OHFV) reconstructed in BEAST using the ORF_het_ data set with 17 sequences (10.245 nt). The purple gradients display three OHFV genotypes/subtypes. The blue horizontal bars are 95% highest posterior density intervals (HPDs) for the main nodes. The asterisk marks the node with posterior probability (pp) < 0.95. All other nodes have pp > 0.95. The vertical dashed line is the moment of the first muskrat release in Western Siberia in 1928 (on the *X*-axis, ~80 years before the most recent sample) [15].

The reconstructed MCC tree had internal nodes with very high pp > 0.95, except for one node with pp = 0.82 (Figure 3). Time to the most recent common ancestors (tMRCA) of OHFV genotypes 1 (117 years; 95% HPD, 92–150) and 3 (62 years; 95% HPD, 42–88) were evaluated with high precision, and their 95% HPD intervals were not overlapped. Therefore, genotype 1 is significantly older than genotype 3.

After cleaning the preliminary data set with TempEst, only one sample of OHFV genotype 2 remained. As a result, we were unable to evaluate tMRCA for this clade. Genotypes 2 and 3, in turn, diverged 461 years ago (95% HPD, 298–667). The MCC tree root had an age of 633 years (95% HPD, 416–896), which characterized OHFV as one of the youngest tick-borne flaviviruses (see Discussion).

**Figure 4:**
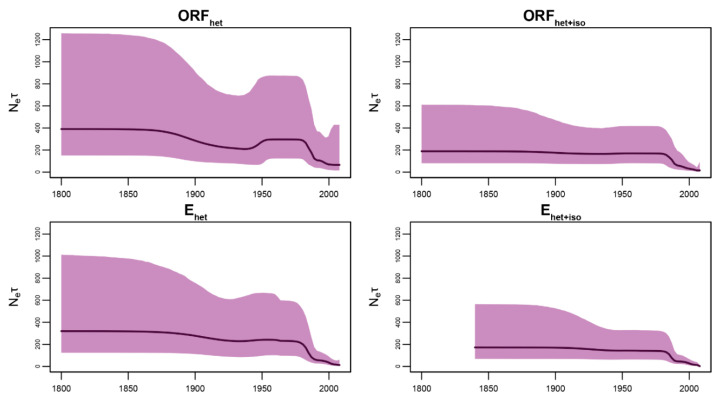
Population dynamics of Omsk hemorrhagic fever virus (OHFV) reconstructed with four heterochronous (het) data sets, where two of them include the isochronous (+iso) samples from Tenis Lake. The purple areas are 95% highest posterior clade density (HPD) intervals, the solid dark lines—median values. The *Y*-axis is relative genetic diversity—N_e_ × τ—where Ne is an effective population size or, in the context of infectious diseases, it is the effective number of infections [14], and τ is the generation time or time between successive infections in transmission chains. The *X*-axis is calendar years.

The past population dynamics of OHFV revealed from the ORF_het_ data set show N_e_ × τ ascending in the 1940s followed by a decline in the 1970s (Figure 4). The E_het_ data set exposed a slight distortion of 95% HPD, and, in the case of data sets with the isochronous components, ascending N_e_ × τ was completely absent in the period of 1940–1970. This is obviously due to the isochronous samples from Tenis Lake, which represented a strong population structure and, thereby, violated the panmixia assumption of the coalescent framework [16]. In addition, the isochronous clade analysis inferred low genetic diversity (Figure 1b) compared with the analysis of the complete OHFV tree (Figure 4), leading to the decrease in the N_e_ × τ trend in the ORF_het+iso_ and E_het+iso_ data sets in the period of 1900–2007.

#### 3.4.2. Isochronous Clade

The isochronous MCC tree was reconstructed in BEAST using 25 OHFV ORF nucleotide sequences from the samples collected in December 2007 (Figure 1b) in Tenis Lake (Omsk region). The analysis demonstrated that the isochronous clade was represented by two lineages that diverged between 79 and 119 years ago. Lineage 1 consisted of 22 closely related viruses that diverged 11–19 years ago. Despite the young age, the viruses carried a sufficient number of SNPs to form multiple reliable clades with pp > 0.95.

The Bayesian skyline reconstruction (Figure 1b) revealed a potential genetic bottleneck with the minimal values of relative genetic diversity (N_e_ × τ ≈ 10) at the end of 2006. The level of N_e_ × τ declined roughly two-fold from the initial values of 20.

## 4. Discussion

**Figure 5:**
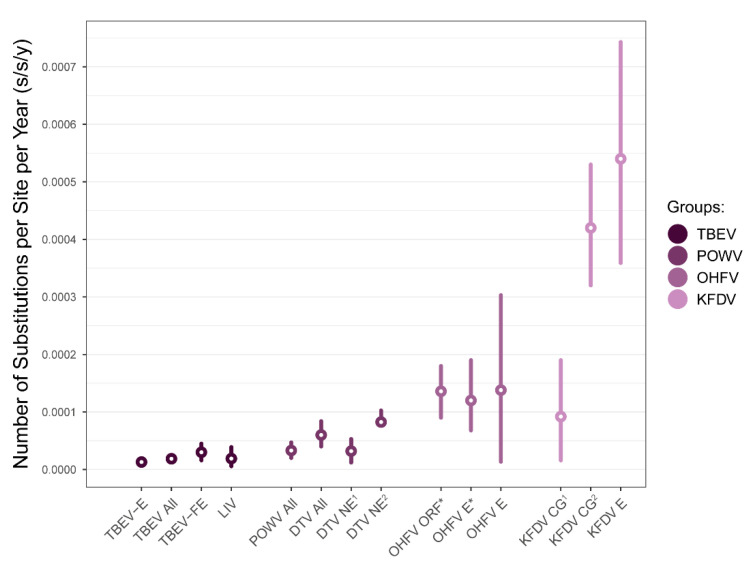
Comparison of substitution rate of tick-borne flaviviruses. Abbreviations: TBEV—tick-borne encephalitis virus; -E—European subtype; -FE—Far-Eastern subtype [19]; LIV—louping-ill virus [25]; POWV All—common clade of Powassan virus [20]; DTV All—common clade of deer tick virus [22]; DTV NE—northeast lineage of deer tick virus [21,22]; OHFV—Omsk hemorrhagic fever virus substitution rate the open reading frame (CG) and envelope (E) genes [3]; KFDV—Kyasanur Forest disease virus substitution rate of a CG [23,24] and E [23] gene. The rates obtained during the current study are marked by asterisks to the right of X-axis labels. Circles represent mean/median values, vertical lines—95% highest posterior density (HPD) intervals.

RNA viruses exhibit substitution rates from 10^−6^ to 10^−3^ s/s/y [17,18]. Our results show that OHFV evolves on the border between magnitudes 10^−5^ and 10^−4^, which is a far higher level in comparison to the most related tick-borne encephalitis virus (TBEV)—the slowest changing tick-borne flavivirus known to date (95% HPD, 1.3–2.4 × 10^−5^ s/s/y) [19]. OHFV values are also higher than those exhibited by another tick-borne flavivirus—Powassan virus (95% HPD, 2.0–4.7 × 10^−5^ s/s/y for the common virus clade [20]; 95% HPDs, 8.23–10.45 × 10^−5^ s/s/y [21] and 1.2–5.3 × 10^−5^ s/s/y [22] for the Northeast clade of the deer tick virus lineage; 95% HPD, 4.0–8.4 × 10^−5^ for the common deer tick virus clade [22]). Among all tick-borne flaviviruses, the substitution rate of OHFV is second only to the Kyasanur Forest disease virus (95% HPDs, 3.2–5.3 × 10^−4^ [23] and 1.6 × 10^−5^–1.9 × 10^−4^ [24]), which also causes hemorrhages in humans; however, it is worth noting that temporal signal in mentioned studies was not evaluated. Hence, OHFV is one of the fast-evolving tick-borne flaviviruses (Figure 5).

The median of the OHFV substitution rate derived in this study (1.36 × 10^−4^ s/s/y) was very close to the previous estimates based on the complete/fragment E gene sequences (1.38 × 10^−4^ s/s/y) [3], wherein the evaluation of temporal signal was not performed. In addition, in our work, the 95% HPD intervals (ORF_het_, 9.1 × 10^−5^–1.8 × 10^−4^ s/s/y) were 3.14-times narrower than was reported by Karan et al. (2014) [3] (1.36 × 10^−5^–3.03 × 10^−4^ s/s/y; Figure 5) and 1.25-times narrower in the case of the widest 95% HPD interval of the E_het+iso_ data set. A more precise substitution rate influenced the width of 95% HPD of OHFV tree root height (95% HPD, 416–896 years before 2007) as opposed to the root height values reported previously, which varied in a wide range (95% HPD, 62–1833 years before 2007), whereas 62 years ago was probably an implausible estimation. Therefore, our study refines knowledge on the occurrence of OHFV, although the 95% HPD interval of root height is still relatively wide.

The temporal signal assessment of OHFV E gene data sets demonstrated that the current number of OHFV E gene sequences contained a very strong temporal structure (Table 2) and could be used for substitution rate evaluation with confidence.

The reconstruction of OHFV population dynamics using the ORF_het_ data set excluding 25 isochronous sequences from Tenis Lake (December 2007) revealed the rise of relative genetic diversity of OHFV in 1940–1970 (Figure 4, the ORF_het_ data set), which coincided with a high number of ticks (*D. reticulatus*) in 1945–1947 and the epizooty of muskrats in 1945–1949 (especially in 1947–1949) in the territory of Western Siberia [1]. Over the 25-year period (1946–1970), 76 muskrat epizooties on the territory of 25 districts of four regions (Tyumen, Kurgan, Omsk, and Novosibirsk regions) were registered. From 1946 to 1952, mass epidemics were also observed. After 1973, there was a decrease in Omsk hemorrhagic fever incidence, followed by an inter-epidemic period that lasted until the last decade of the 20th century. The short activity of the foci in the early 1990s was replaced by a decrease in a number of cases of the disease. Thus, since 2002, new cases of Omsk hemorrhagic fever were not observed [1]. This declining trend at the end of the 20th century was also inferred during our Bayesian skyline reconstruction. But it should be noted that despite excluding 25 samples collected at Tenis Lake, the population structure in the data is still obvious [3]. This potentially leads to a biasing coalescent inference known as the confounding effect, leading to a false signal of a sharp decline in relative genetic diversity [13].

Molecular dating of the 25 isochronous OHFV samples from Tenis Lake revealed that the clade diverged around 97 years ago (95% HPD, 79–119) (Figure 1b). In total, 22 samples of the lineage 1 form a subcluster with a younger divergence time of 15 years ago (95% HPD, 11–19). Some of the subclusters with low pp exhibit short inner branches relative to external ones, which is the pattern of exponential growth of viral population size [26]. In other words, a large proportion of the observed genetic diversity of the OHFV Tenis Lake population comprising persistent lineages and distinct SNPs were formed for a relatively short time, which indicated the evolutionary and epidemic potential of OHFV. Such local foci are considered to be likely sources of spillover into humans. Furthermore, the evolutionary timescale of OHFV is different from the most related TBEV due to a faster substitution rate (10^−4^ and 10^−5^ magnitudes, respectively); the finer timescale of OHFV is mainly manifested as younger divergence times (<150 years ago) of main nodes (Figure 1b and Figure 3) in comparison with the hundreds of years for TBEV [19,27]. Furthermore, we detected a bottleneck at the end of 2006 (i.e., one year before the sample collection), which could have been induced by unclear ecological factors (phase of N_e_ × τ decline in 2005–2006) or the result of recent immigration events (N_e_ × τ increasing in 2006–2007) followed by the infestations of previously uninfected hosts. This local trend is opposite to the Bayesian skyline inferred from the data set comprising the OHFV samples from the different geographical locations.

Previously, three hypotheses on the OHFV origin in the Siberia territory were proposed [1]. The first hypothesis is the spread of the virus from the USA and Canada due to the *O. zibethicus* introduction into the Eurasia territory [28], with the peak period in 1935–1939, when 4340 muskrats were released [15]. The second hypothesis is that OHFV is a native virus species. The third hypothesis is the OHFV introduction from India, where the Kyasanur Forest disease virus with similar hemorrhagic manifestation circulates. Our results confirmed the second one: the MRCA of all OHFV strains diverged hundreds of years before the muskrat introduction into Siberia (Figure 3) in the 20th century, which coincided with the previous results based on the first OHFV divergence dates [3]. Furthermore, of all tick-borne flaviviruses, only Powassan virus was registered on the territory of North America, whereas OHFV, in turn, was detected outside Russia only in the Republic of Kazakhstan [4]—which is the southern part of ranges of *D. marginatus* and *D. reticulatus* ticks [29] in Asia. Another aspect is that the reservoir host range of OHFV is not limited by *O. zibethicus*. In some rodent species, such as *Microtus oeconomus* or *Arvicola amphibius*, OHFV induce chronic disease that better facilitates maintaining a virus in a two-host system (vertebrates and invertebrates) than an acute form of the disease with a fatal outcome, like in *O. zibethicus*. We speculate that the drastic rise of OHFV incidence in humans and muskrats in the middle of the 20th century, which has been reflected in the reconstruction of OHFV past population dynamics, can be a consequence of the contact of *O. zibethicus*—susceptible and unadapted hosts—with native OHFV but not the emergence of a new virus in the territory of Siberia. Last, the counterargument to the hypothesis on the OHFV introduction from India is the most related phylogenetic relationship between OHFV and TBEV. The similar hemorrhagic manifestations of OHFV and the Kyasanur Forest disease virus are due to the same genomic determinants in the polyprotein gene [30].

Even though OHFV and TBEV belong to two different genetic lineages, the ranges of the species are overlapped. At the same time, these viruses are mainly transmitted by ticks from two different genera (*Dermacentor* and *Ixodes*, respectively), due to which two species reduce competition for a vector. However, vector-specificity cannot support a hypothesis on the coevolution of viruses and ticks since the divergence of *Dermacentor* and *Ixodes* genera occurred dozens of million years ago [31], which exceeds the evolutionary timescale of flaviviruses [32]. In other words, biological or ecological factors underlying the OHFV and TBEV divergence remains unclear. The divergence time between OHFV and TBEV assessed using amino acid sequences of ORF with removing ambiguous regions is about 2000 years ago, but these estimates contradict ones based on nucleotide data supported by temporal structure evaluation, which infer that the TBEV emergence date is about 11,000 years ago [19]. Thus, with some confidence, actual data allow us to conclude that the divergence between OHFV and TBEV occurred more than 11,000 years ago, i.e., before the Holocene. Further investigations, including the assessment of the phylodynamic threshold of tick-borne flaviviruses, are required.

To summarize, notwithstanding the absence of Omsk hemorrhagic fever cases in humans and mass epizooties in muskrats, OHFV continues to evolve in local and, apparently, a few natural foci, which poses a potential threat to public health.

## Figures and Tables

**Table 1:** Determination coefficients of the root-to-tip regression and substitution rate values for the Omsk hemorrhagic fever virus (OHFV) data sets.

Data Set ^1^	SW, Years ^2^	Sample Size	R^2^	Number of PI Sites ^3^	Substitution Rate, s/s/y ^4^	Width of 95% HPD (Relative Error) ^5^	
						Rate	Root
ORF_het_	61	17	0.42	1396	1.3 × 10^−4^	9.2 × 10^−5^ (49%)	480 (53%)
ORF_het+iso_	61	39	0.62	1481	1.3 × 10^−4^	1.3 × 10^−4^ (59%)	579 (63%)
E_het_	61	49	0.11	223	1.2 × 10^−4^	1.2 × 10^−4^ (64%)	787 (68%)
E_het+iso_	61	73	0.21	228	1.9 × 10^−4^	2.3 × 10^−4^ (71%)	597 (77%)

^1^ Abbreviations in subscripts: het—heterochronous data set, het+iso—heterochronous data set with the isochronous samples collected from Tenis Lake (Omsk Region) at one time point (2007); ^2^ SW—sampling window (time between the most recent and oldest samples in a data set); ^3^ number of parsimony-informative (PI) sites divided by the number of sites (alignment length). PI sites were estimated in IQTREE; ^4^ s/s/y—substitutions per site per year (mean values); ^5^ width of 95% highest posterior density (HPD) intervals for substitution rate (substitutions per site per year) and root height (years) assessed in BEAST2. Relative error was estimated to be (HPD_right_ − HPD_left_)/HPD_right_ × 100, where HPD_right_ is a HPD right border value, HPD_left_ is a HPD left border value.

**Table 2:** Results of Bayesian evaluation of temporal signal.

Data Set ^1^	Clock ^2^	Log Marginal Likelihood ^3^	Log Bayes Factor ^3^
ORF_het_	SC_dates_	−26,066.03	4.17
	**UCLD_dates_**	**−26,061.86**	0
	SC_none_	−26,116.01	54.15
	UCLD_none_	−26,072.29	10.43
ORF_het+iso_	SC_dates_	−23,624.41	4.94
	**UCLD_dates_**	**−23,619.47**	0
	SC_none_	−23,657.86	38.39
	UCLD_none_	−23,638.48	19.01
E_het_	SC_dates_	−4200.11	3.72
	**UCLD_dates_**	**−4196.39**	0
	SC_none_	−4219.24	22.85
	UCLD_none_	−4217.9	21.51
E_het+iso_	SC_dates_	−4411.16	12.04
	**UCLD_dates_**	**−4399.12**	0
	SC_none_	−4449.96	50.84
	UCLD_none_	−4448.55	49.43

^1^ Abbreviations in subscripts: het—heterochronous data set, het+iso—heterochronous data set with the isochronous samples collected from Tenis Lake (Omsk Region) at one time point (2007); ^2^ Molecular clock models: SC—strict clocks, UCLD—uncorrelated relaxed clocks with underlying lognormal prior distribution of substitution rates varying between tree branches; ^3^ best-performing model has a log Bayes factor value of 0.

## Data Availability

BEAST2 xml files and logs for each data set can be downloaded from the following link: https://doi.org/10.6084/m9.figshare.22880288.v1.

## Supplementary Materials

The following supporting information can be downloaded at: https://www.mdpi.com/article/10.3390/v15071576/s1, Figure S1: Regression of the OHFV genetic distance from the root to each of the tips as a function of their sampling times estimated in TempEst.
