# Supplementary figures and images for: Inferring Evolutionary Timescale of Omsk Hemorrhagic Fever Virus
Source: Viruses. 2023 Jul 19;15(7):1576. doi: 10.3390/v15071576 (PMC10385366; doi:10.3390/v15071576)

**CGhet**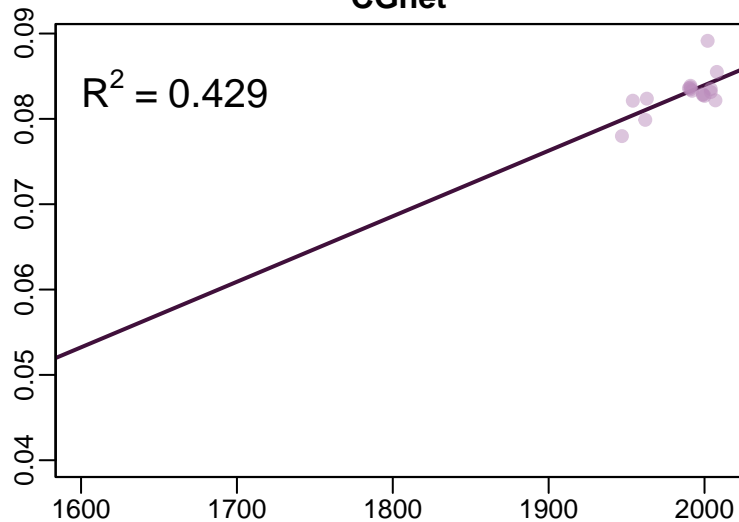**CGhet+iso**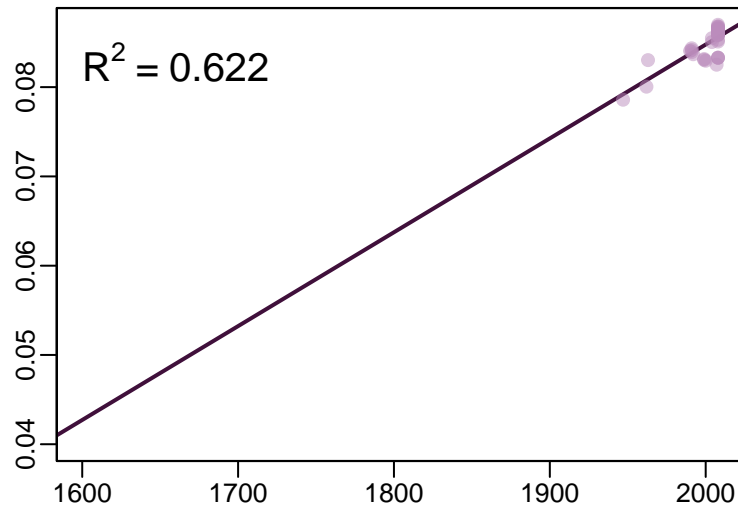**Ehet**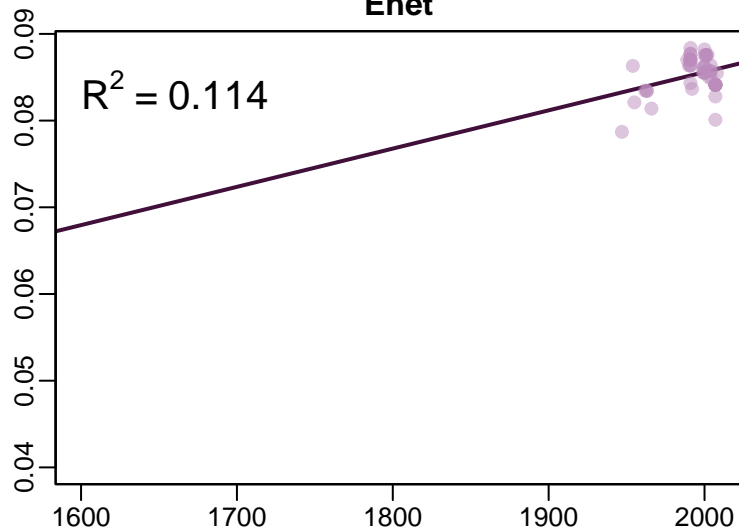**Ehet+iso**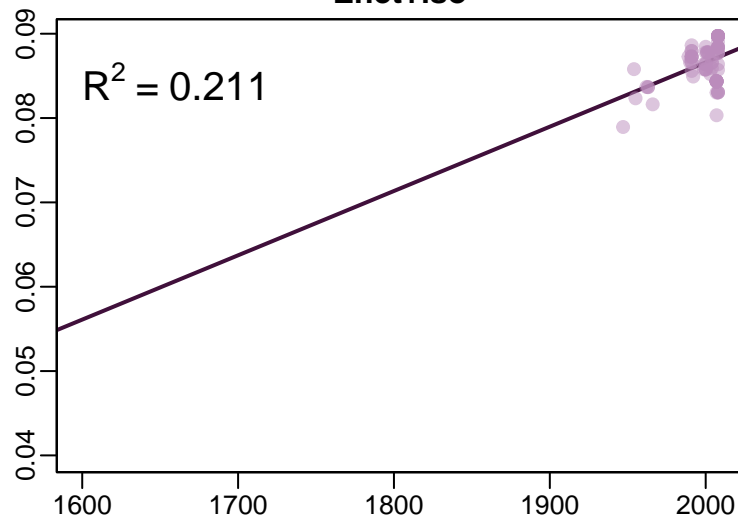

Supplement: Supplementary file 1 [file viruses-15-01576-s001.zip › Figure S1.pdf]
